# Low pH Does Not Impact Reproductive Success but Leads to Negative Carry‐Over Effects Between Parents and Larvae in a Mediterranean Gastropod

**DOI:** 10.1002/ece3.72254

**Published:** 2025-10-02

**Authors:** Sanja Grđan, Sam Dupont, Luka Glamuzina, Ana Bratoš Cetinić

**Affiliations:** ^1^ Department of Applied Ecology University of Dubrovnik Dubrovnik Croatia; ^2^ Department of Biological and Environmental Sciences University of Gothenburg Fiskebäckskil Sweden; ^3^ IAEA Marine Environment Laboratories Radioecology Laboratory Monaco Monaco

**Keywords:** Adriatic Sea, carryover effect, intracapsular development, ocean acidification, parental exposure, reproduction

## Abstract

Understanding how marine organisms respond to ocean acidification across all life stages is essential for assessing the future resilience of ecosystems. We investigated the effects of long‐term exposure to low pH conditions (pH_T_ ranging from 7.95 to 7.22) on the reproductive traits and intracapsular development of *Hexaplex trunculus*, a predatory Mediterranean gastropod. Spawning success, fecundity, and capsule morphology were not affected by pH. However, larval development was significantly impaired at pH_T_ lower than 7.51, with observed delayed development and fewer larvae developing successfully to the hatchling stage. Cross‐transplantation of spawns between pHs indicated a negative carryover effect of parental exposure to low pH on larval development, although this was partially reversible when spawns were transferred back to the ambient pH. Notably, we observed inter‐individual variability in larval growth, suggesting that phenotypic plasticity or genotype‐specific tolerance may play a role in moderating sensitivity to future ocean acidification. Our study highlights the importance of considering parental exposure, natural pH variability, and within‐population variation when assessing species responses to global drivers.

## Graphical abstract

We investigated the long‐term effects of ocean acidification on reproduction and early development in the Mediterranean gastropod *Hexaplex trunculus*. While low pH did not affect spawning or reproductive output, it significantly delayed larval development and reduced the likelihood of reaching hatching, especially when parents were preconditioned to acidified conditions. Our findings highlight the importance of parental exposure and intraspecific variability in assessing species responses to future ocean acidification.

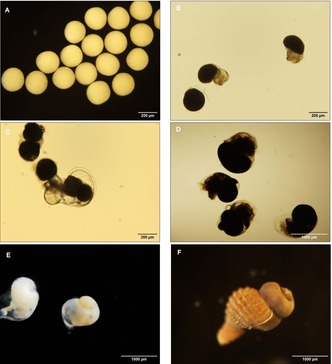

## 1 Introduction

Marine ecosystems have been increasingly pressured over the past decades, with ocean acidification (OA) emerging as one of the main drivers of global changes. Since the Industrial Revolution, atmospheric CO_2_ levels have increased by approximately 50%, altering seawater carbonate chemistry and lowering the average pH of the surface ocean by 0.11 units (Doney et al. 2009; Friedlingstein et al. 2022). Projections indicate a further decrease of 0.08–0.37 units by the end of the century (Cooley et al. 2022). These changes can have complex and species‐specific physiological effects, making it essential to study processes critical to species fitness to better understand potential vulnerabilities and adaptive capacities under future ocean conditions (Hurd et al. 2020).

Reproductive performance is a central determinant of population resilience and should be evaluated across multiple traits ‐ including gamete production, fecundity, capsule morphology, and hatching success—rather than single endpoints (Padilla‐Gamiño et al. 2022). Previous studies have shown highly variable responses to low pH among species ‐ some reporting significant effects on reproductive traits (Xu et al. 2016; Conradi et al. 2019; Rossin et al. 2019; Marčeta et al. 2020), while others report no effect (Reed et al. 2021; Uthicke et al. 2021). In addition to other parameters, the duration of exposure plays a decisive role in shaping the magnitude of low pH effects. In some species, long‐term acclimation can mitigate negative short‐term effects but still result in negative carryover effects for offspring (Dupont et al. 2013). Others show changes in reproductive traits, such as altered egg size or failure to produce viable larvae, despite apparently successful gametogenesis (Glass et al. 2023; Pansch et al. 2018).

Larval stages are particularly vulnerable to ocean acidification (e.g., Bergman et al. 2018; Byrne and Hernández 2020), as they lack fully developed compensatory mechanisms for pH homeostasis and require more energy for development and basic life functions compared to adult stages (Stumpp et al. 2011; Lee et al. 2020).

Negative effects of stress on larval development can occur after short exposure during larval stages, as demonstrated in gastropods where brief stress led to reduced post‐metamorphic performance (Montroy et al. 2016). Similarly, slower growth rates have been observed in the gastropod *Crepidula fornicata* larvae after exposure to low pH, although without an impact on survival (Pechenik 2018). Additionally, recent work on *Crepidula fornicata* further demonstrates that responses to ocean acidification cannot be fully understood by examining a single life stage in isolation. Larval exposure to reduced pH not only impaired larval growth but also carried over into the juvenile stage, where growth was further reduced even when larval development had appeared unaffected (Bogan et al. 2019). These findings highlight the importance of considering trans‐life‐cycle effects, where early exposure to stress interacts with other factors such as nutrition and produces latent consequences that only emerge later in development. While most invertebrate species exhibit some kind of negative effects on early stages under low pH (e.g., Pechenik 2018), some do show positive effects, such as increased growth rates of bay scallop *Argopecten irradians* (Lamarck, 1819) (Gobler and Talmage 2012) or no observed effect at all (e.g., Bailey et al. 2017). Physiological responses can vary even among different populations of the same species. For example, long‐term experiments (6 months) on adult sea urchins *Paracentrotus lividus* (Lamarck, 1816) from different environments showed differences in the magnitude of response where both populations eventually acclimated, but sea urchins from a more variable environment appeared to acclimate faster to low pH (Asnicar et al. 2021). A recent study by Vargas et al. (2022) revealed that the impact of scenarios used in ocean acidification experiments on marine organisms depends on the deviation from the upper *p*CO_2_ level experienced by local populations and highlights the importance of considering the present *p*CO_2_ natural variability for a given population.

Although mollusks are among the most studied taxa in OA research, work has focused mainly on bivalves, while gastropods remain comparatively overlooked despite their ecological and economic significance. Gastropods exhibit diverse responses to OA, including impaired larval development, reduced growth and calcification, altered feeding, and changes in metabolism (Noisette et al. 2015; Carey et al. 2016; Pechenik et al. 2019; Young et al. 2019; Barclay et al. 2020). Given their ecological roles as both grazers and predators, and their influence on community dynamics, key species responses should be further investigated in habitats of interest. Within the Mediterranean, predatory gastropods such as the banded‐dye murex, *Hexaplex trunculus* (Linnaeus 1758), play a key ecological role as bivalve predators and influence benthic community dynamics (Rilov et al. 2004; Peharda and Morton 2006; Güler and Lȍk 2018). While its reproductive cycle and embryonic development have been previously described (Vasconcelos et al. 2004; Lahbib et al. 2010; Güler and Lȍk 2014), little is known about its capacity to reproduce successfully under future low pH conditions. There are a few experimental studies on the impact of ocean acidification on banded‐dye murex addressing feeding behavior (Chatzinikolaou et al. 2019, Grđan et al. 2022), shell growth rate (Grđan et al. 2023), morphological traits, and differences in response between males and females (Grđan et al. 2025a, 2025b), indicating trade‐offs while reallocating energy between different life processes.

The population of banded‐dye murex used in this study originates from the Mali Ston Bay (southeastern Adriatic Sea), an area with significant natural fluctuations of environmental factors, influenced by freshwater inflow, temperature seasonality, and biological activity (Pećarević et al. 2020). Natural pH variability within the species' native habitat is considered to assess the potential for resilience or vulnerability. This long‐term study aims to assess the effect of prolonged exposure to a range of pH on reproductive traits of *H. trunculus*, focusing on spawning, fecundity, capsule morphology, intracapsular development, and hatching success while also evaluating the carryover effect of parental exposure on the embryo's sensitivity to pH. By studying the reproductive output and offspring performance under different pH scenarios, this study provides new insights into the resilience or potential vulnerability of banded‐dye murex under future ocean acidification.

## 2 Materials and Methods

### 2.1 Sample Collection and Experiment Set‐Up

Full details of the sampling and experimental setup have been described in Grđan et al. (2023). In brief, mature adult banded‐dye murexes were collected from Bistrina Bay, a part of Mali Ston Bay in the southeastern Adriatic Sea, and transferred to the nearby Laboratory for Mariculture, University of Dubrovnik. Gastropods were cleaned from fouling organisms and immersed in an anesthetic solution of magnesium chloride hexahydrate (MgCl_2_ × 6H_2_O) to relax their strong pedal muscle (Gibbs 1999). Sexes were determined by the presence or absence of a penis behind the right tentacle in the cephalic region. Ten females and 30 randomly chosen individuals per pH (40 per pH, 360 in total) were marked with a numbered bee tag glued to the shell. Nine flow‐through tanks (volume 130 L) were filled with seawater pumped from the adjacent bay, filtered, and sterilized. Prior to the start of the experiment, all gastropods were maintained under ambient seawater conditions in flow‐through tanks and fed ad libitum with the Mediterranean mussel *Mytilus galloprovincialis* Lamarck 1819 for a two‐week acclimation period. This ensured recovery from collection and handling stress before exposure to the pH treatments. To reflect natural seasonal cues for reproduction, temperature and salinity were not artificially adjusted but allowed to fluctuate within the natural ambient range. This was intentional, as temperature is a key driver of gametogenesis and spawning in the banded‐dye murex.

pH was manipulated independently in each tank by continuous injection of pure CO_2_, regulated with a pH controller (Milwaukee MC122) to maintain stable setpoints. One tank served as an untreated control. In total, nine pH levels were maintained, ranging from 7.95 (control) to 7.22, with 7.95–7.78 representing the natural variability measured in Bistrina Bay. To ensure treatment stability, pH was measured on the total scale every second day, calibrated with TRIS buffer (Scripps Laboratory, batch T37). Total alkalinity (TA, μmol kg^−1^) was determined biweekly using manual two‐point open‐cell titration with 0.1 M HCl (Dickson et al. 2007), and carbonate chemistry parameters were calculated accordingly.

Potential confounding factors were closely monitored. Temperature, salinity, and dissolved oxygen concentrations were recorded daily using handheld probes (YSI Pro 30 and Oxygen Handy Polaris). Values remained within natural seasonal ranges and did not differ systematically among treatments. Thus, differences among treatments can be attributed to pH manipulation rather than to uncontrolled variability in other seawater parameters. The experiment started on August 24th, 2020.

### 2.2 Reproductive Success and Intracapsular Development

Measured key aspects of reproductive success for the banded‐dye murex in this study were the proportion of females that spawned, the number of capsules per spawn, egg number per capsule, and the proportion of spawns reaching hatching. The expected spawning time of *H. trunculus* is late spring (Vasconcelos et al. 2004), when the sea temperature begins to rise. To obtain individual spawns, marked females were separated into individual containers at the beginning of May 2021 (250 days of exposure). Each female was transferred to a 5‐L plastic canister cut open at the top and covered with a net to prevent escapes. In addition, longitudinal openings were cut three centimeters from the bottom of the containers on opposite sides to allow water to flow through the container. Until the start of spawning, females were fed Mediterranean mussel, *Mytilus galloprovincialis* . The start and duration of spawning were recorded for each female. After the completion of spawning, females were taken out from the containers and placed back in the tank with the other individuals. Spawn was left in the container. The number of females spawning was recorded for each pH treatment. Immediately after spawning, 10 randomly selected capsules from each spawn were measured with a digital caliper (precision 0.01 mm) for length (cl, mm), width (cw, mm), and thickness (ct, mm), with the length being the greatest distance between the basal membrane and the apex, width the greatest distance between lateral edges at right angles to length, and thickness the greatest distance from convex side to concave side at right angles to length and width (D'Asaro 1986). Five capsules were carefully opened with a scalpel; eggs were emptied onto a microscope slide and counted under a stereo microscope (Olympus SZ40).

Egg viability was tracked through successive sampling of spawned capsules and measurements of embryos. The method for monitoring intracapsular development was modified following previous studies by Vasconcelos et al. (2004), Lahbib et al. (2010), and Güler and Lȍk (2014). Four days after spawning, between two and five capsules were carefully removed from each spawn. Fertilized eggs were emptied onto a microscope slide and photographed with a microscope digital camera (Olympus DP72) under a light microscope (Olympus BX51). The diameter of a minimum of 50 eggs from each capsule was measured with the software Fiji. To determine the stage of intracapsular embryonic development, five capsules were randomly sampled from each spawn at least four times until hatching, at intervals of approximately 5 days. Although more frequent sampling could have provided finer temporal resolution, our priority was to preserve a sufficient number of viable capsules until the end of the experiment. Because each sampling carried a risk of damaging neighboring capsules, a conservative sampling frequency was chosen to minimize disturbance. The capsules were preserved in a 4% solution of formaldehyde in seawater for further analysis. Each capsule was carefully opened with a scalpel and emptied onto a slide. The embryos were photographed under a light microscope and the length was measured using the Fiji software. The developmental stage was determined based on the characteristic structures. The first larval stage, the trochophore, was determined by the fine cilia on the anterior side (Lahbib et al. 2010). The first characteristic structure of the veliger stage is the development of a short, bi‐lobed velum, eyes, and visceral mass, indicating an early veliger (Lahbib et al. 2010; Güler and Lȍk 2014). Further veliger stage was determined by shell formation and more pronounced velar lobes (Vasconcelos et al. 2004). The development of the foot and the large four‐lobed velum indicated pediveliger larvae. At the end of intracapsular development, the velum began to degenerate and the shell became pigmented yellow‐brown. The hatchlings pierced the fine membrane covering the capsular opening and crawled outside (Vasconcelos et al. 2004; Lahbib et al. 2010).

### 2.3 Carryover Effect

**FIGURE 1:**
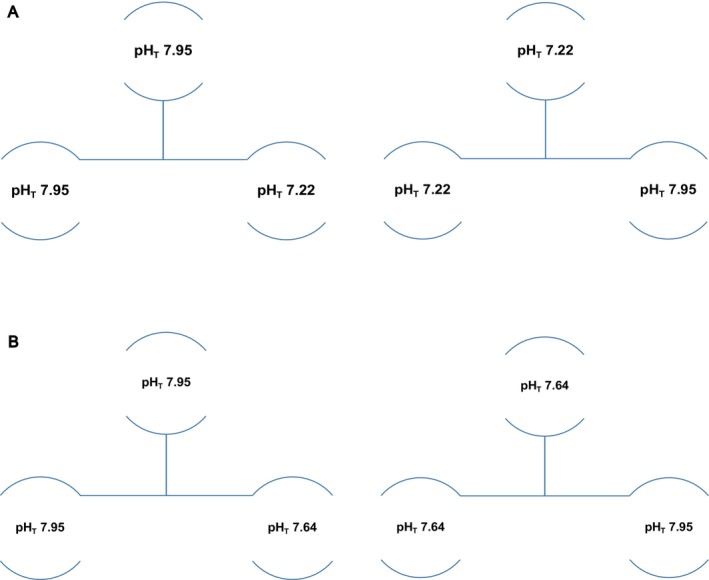
Cross transplantation of *Hexaplex trunculus* spawns for evaluation of carryover effect (A) between pHT 7.95 & 7.22 and (B) between pHT 7.95 & 7.64.

To evaluate the carryover effect of parental exposure on the embryos' sensitivity to pH, spawns were transferred to different pHs following the scheme presented in Figure 1. Spawns were selected based on their size and accessibility. Additionally, for the cross‐transplantation between pHs 7.95 and 7.64, a spawn from pHT 7.94 (manipulated treatment, female 6) was chosen due to a lack of suitable spawns in 7.95 (unmanipulated treatment). After females completed the spawning, the selected spawns were carefully separated with a scalpel and cut in half. One‐half of the spawn was returned to the pH_T_ from which it had been removed, and the other half was placed in the designated pH_T_. At a minimum of four times over the course of the intracapsular development, two capsules were carefully removed from transplants and placed in 4% formaldehyde for further analysis (measurement of embryo size and developmental stages).

### 2.4 Statistical Analyses

Statistical analyses were performed using SPSS Statistics v.26. The binary logistic regression model was applied to determine if pH had a significant effect on the likelihood of spawning and on the likelihood of reaching a developmental stage (when applicable), with the regression coefficient estimated (*β*) interpreted as a predicted change in log odds for every single unit increase of pH. The relationships between pH_T_ and capsule length, width, and thickness, average number of eggs per capsule, number of spawned capsules, and egg diameter per spawn were tested with simple linear regression. The mean difference in the average developmental stage length among pH_T_ was tested with one‐way ANOVA, and the relationship between the day post spawning when a respective developmental stage was reached and pH_T_ was tested with simple linear regression (SLR). Intracapsular growth rate for each transplanted spawn was calculated from the log‐linear relationship between developmental stage length and developmental time (μm log day^−1^). After log linearization of the data, embryo length between transplants was compared with ANCOVA with developmental time as a covariate. Prior to analysis, the data were tested for normality of residuals with a Q–Q plot or Shapiro–Wilk's test, and for the equality of variances with Levene's test. All data met the assumptions. The threshold for significance was set at *p* < 0.05. When a significant effect was observed, a post hoc Tukey pairwise comparison was applied with Bonferroni correction for multiple comparisons. Estimated marginal means (EMMs ± SE) obtained from the model were used to further investigate the trend of the relationship between pH_T_ and the dependent variable.

## 3 Results

### 3.1 Seawater Parameters

Measurements of temperature and salinity, and carbonate chemistry data are available in the PANAGEA Data Publisher Repository (Grđan et al. 2024). Temperature and salinity varied with seasonal changes, and dissolved oxygen concentration was above 6.28 mg L^−1^ O_2_. Measured and calculated carbonate chemistry parameters are listed in Table 1. pH_T_ in the unmanipulated treatment fluctuated between 7.75 and 8.05.

**TABLE 1:** Carbonate chemistry parameters are presented as Mean ± SD. Measured: Seawater pH on a total scale (pH_T_) and total alkalinity (TA; mmol kg^−1^). Calculated: CO_2_ partial pressure (*p*CO_2_; μatm), calcite and aragonite saturation states (Ω_Ca_ and Ω_Ar_, respectively) (Grđan et al. 2023).

pH_T_	TA (mmol kg^−1^)	*p*CO_2_ (μatm)	Ω_Ca_	Ω_Ar_
7.95 ± 0.07	2976 ± 22	692 ± 18	3.9 ± 0.8	2.5 ± 0.6
7.95 ± 0.08	2950 ± 18	698 ± 19	3.9 ± 0.9	2.5 ± 0.6
7.87 ± 0.08	2940 ± 20	809 ± 77	3.3 ± 0.4	2.1 ± 0.3
7.76 ± 0.07	2955 ± 20	1064 ± 99	2.7 ± 0.4	1.7 ± 0.3
7.64 ± 0.07	2935 ± 21	1335 ± 12	2.2 ± 0.3	1.4 ± 0.2
7.51 ± 0.07	2917 ± 21	1759 ± 17	1.7 ± 0.2	1.1 ± 0.2
7.42 ± 0.07	2946 ± 18	2187 ± 18	1.4 ± 0.9	0.9 ± 0.1
7.33 ± 0.06	2891 ± 21	2601 ± 24	1.2 ± 0.2	0.7 ± 01
7.22 ± 0.07	2851 ± 18	3221 ± 25	0.9 ± 0.6	0.6 ± 0.1

### 3.2 Reproduction and Intracapsular Development

**FIGURE 2:**
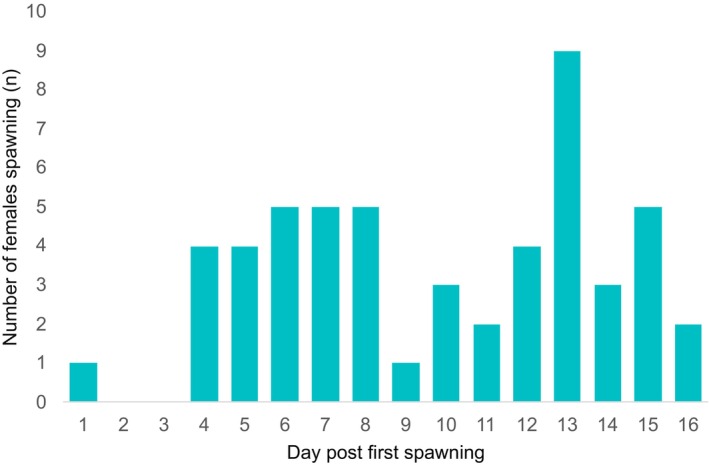
Number of tagged females of *Hexaplex trunculus* spawning on each day during the overall spawning duration. The day when the first spawning event was recorded was denoted as Day 1.

Temperature reached above 20°C on 22 May 2021, which triggered spawning. The first spawning happened on 31 May 2021 (pH_T_ 7.94) and was denoted as Day 1. Next, females spawned three days later, followed by two to six females per day. The peak of the spawning was on Day 13 with nine females spawning. The last spawning was recorded on the 16th day (Figure 2).

pH_T_ level had no significant impact on the likelihood of females spawning (BLR, *χ* ^2^(1) = 2.168, *p* = 0.141). The average duration of spawning for all pH_T_ treatments was 2.92 ± 0.87 days, with a maximum of 5 days (pH_T_ 7.22) and a minimum of 1 day (pH_T_ 7.51). pHT level had no significant effect on the number of spawned capsules (SLR, *R* ^2^ = 0.001, *F*(1, 52) = 0.003, *p* = 0.953) (Table 2).

**TABLE 2:** Number of females of banded‐dye murex, *Hexaplex trunculus*, spawning (*N*), mean spawning duration in days and the number of spawned capsules (*n*) per pH_T_ (mean ± standard deviation).

pH_T_	N females spawning (out of 10)	Mean duration (days)	*n* capsules
7.95 ± 0.07	7	3.20 ± 1.30	196 ± 60.78
7.95 ± 0.08	7	2.25 ± 0.50	244 ± 97.87
7.87 ± 0.08	6	3.33 ± 0.81	222 ± 100.87
7.76 ± 0.07	6	1.80 ± 0.83	272 ± 110.85
7.64 ± 0.07	7	3.00 ± 0.81	218 ± 89.68
7.51 ± 0.07	5	3.33 ± 0.51	197 ± 54.30
7.42 ± 0.07	6	2.83 ± 0.98	234 ± 57.45
7.33 ± 0.06	4	3.29 ± 0.48	266 ± 132.84
7.22 ± 0.07	5	2.86 ± 0.69	210 ± 55.67
Average	5.9 ± 1.05	2.92 ± 0.87	228 ± 83.38

Ten capsules from each spawn (530 in total) were measured for length, width, and thickness, with no effect of pH (SLR, *R* ^2^ = 0.014, *F*(1, 529) = 5.729, *p* = 0.017; *R* ^2^ = 0.022, *F*(1, 529) = 9.306, *p* = 0.002; *R* ^2^ = 0.008, *F*(1, 529) = 4.122, *p* = 0.043, respectively). The mean measured capsule length was 4.76 ± 0.54 mm, width was 4.13 ± 0.53 mm, and thickness was 1.53 ± 0.26 mm. Five capsules from each spawn were carefully opened to count the number of eggs (265 in total). pH had no significant effect on the mean number of eggs per spawn (SLR, *R* ^2^ = 0.007, *F*(1, 52) = 0.364, *p* = 0.549). The mean length, width, and thickness of capsules, as well as the number of eggs per capsule in each pH, are presented in Table 3.

**TABLE 3:** *Hexaplex trunculus* capsule (*n* = 5) length (cl, mm), width (cw, mm), and thickness (ct, mm), and the number of eggs per capsule (n eggs) per pH_T_ (mean ± standard deviation).

pH_T_	cl (mm)	cw (mm)	ct (mm)	*n* eggs
7.95 ± 0.07	4.57 ± 0.49	3.88 ± 0.36	1.52 ± 0.26	255 ± 71
7.95 ± 0.08	4.78 ± 0.43	4.02 ± 0.51	1.52 ± 0.22	251 ± 85
7.87 ± 0.08	4.93 ± 0.48	4.21 ± 0.49	1.45 ± 0.20	287 ± 33
7.76 ± 0.07	4.35 ± 0.54	4.13 ± 0.45	1.51 ± 0.29	291 ± 80
7.64 ± 0.07	4.66 ± 0.52	3.96 ± 0.40	1.49 ± 0.23	250 ± 86
7.51 ± 0.07	4.89 ± 0.50	3.94 ± 0.46	1.45 ± 0.29	269 ± 22
7.42 ± 0.07	4.70 ± 0.44	4.27 ± 0.65	1.58 ± 0.27	274 ± 84
7.33 ± 0.06	4.97 ± 0.54	4.37 ± 0.49	1.57 ± 0.28	274 ± 75
7.22 ± 0.07	4.61 ± 0.62	3.93 ± 0.59	1.56 ± 0.26	279 ± 115
Average	4.76 ± 0.54	4.13 ± 0.53	1.53 ± 0.26	271 ± 62

**FIGURE 3:**
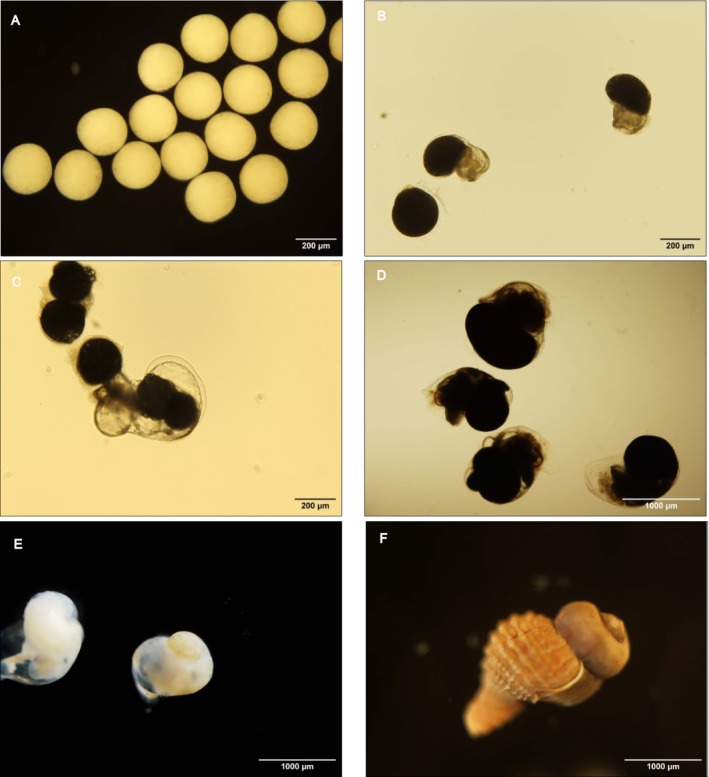
Stages of *Hexaplex trunculus* intracapsular development, pHT 7.95: (A) fertilized eggs, (B) trochophore, (C) early veliger, (D) veliger, (E) pediveliger, (F) hatchling.

Intracapsular embryonic development started with fertilized eggs, followed by the development of trochophore, early veliger, veliger, pediveliger larvae, and hatchling stage (Figure 3).

**FIGURE 4:**
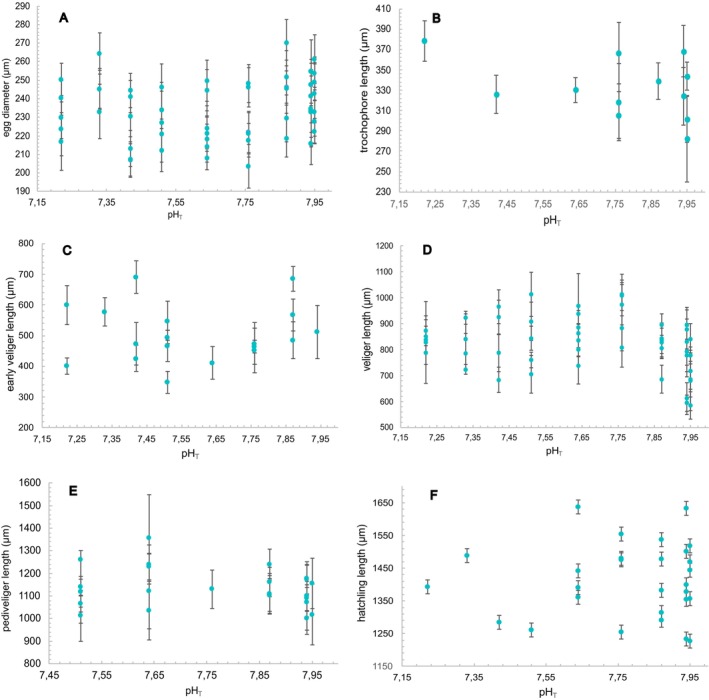
Relationship between (A) egg diameter (μm), (B) trochophore, (C) early veliger length, (D) veliger, (E) pediveliger and (F) hatchling length (μm) of *Hexaplex trunculus* and pHT. Each dot represents the mean length ± SE (μm) per spawn.

Four days after spawning, capsules were sampled to measure the diameter of the fertilized eggs. For each spawn, between two and five capsules were carefully opened, and eggs were photographed under the microscope. A diameter of a minimum of 100 eggs per spawn was measured with Fiji software. Embryos that already started cell divisions were not measured. pH had no significant effect on the average egg diameter per spawn (SLR, *R* ^2^ = 0.029, *F*(1, 52) = 1.525, *p* = 0.222; Figure 4).

**FIGURE 5:**
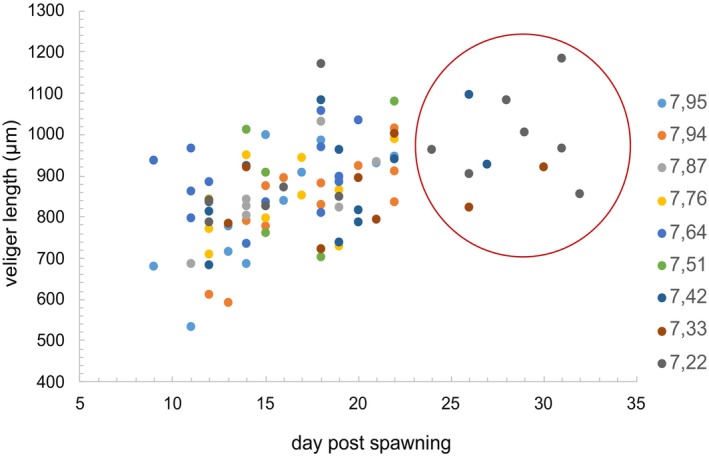
Relationship between the veliger length and the day post‐spawning (DPS) of *Hexaplex trunculus* for each pHT (colored dots) indicating longer veliger development time for pHT 7.51–7.22 (DPS 22–32, red circle).

After the initial sampling of the newly deposited capsules from each spawn, further capsules were randomly sampled at a minimum of four times over the duration of their intracapsular development, except for the spawns where development was arrested. Spawns were not sampled on the same day post‐spawning. In several spawns, embryos developed into the next stage between the two capsule samplings; therefore, not all developmental stages were measured for each spawn. However, this does not indicate that the spawn did not go through that stage. For each recorded developmental stage, all individuals were measured, and the corresponding day post‐spawning (DPS) was noted to calculate the average timing of stage appearance. In the samples between the fifth‐ and ninth‐day post‐spawning, trochophore larvae were recorded for every pH_T_, except for 7.51 and 7.33. Trochophore larvae were reached on average on 6.91 ± 1.16 DPS with a mean trochophore length of 331.94 ± 29.02 μm. pH had no significant effect on the time taken for embryos to reach the trochophore stage (SLR, *R* ^2^ = 0.038, *F*(1, 11) = 0.395, *p* = 0.544). There was no difference in the average trochophore length among pH treatments (one‐way ANOVA, *F*(6, 35) = 1.632, *p* = 0.174; Figure 4B). The early veliger stage was recorded in the samples between the ninth‐ and eleventh DPS (9.50 ± 1.20 DPS) in every pH_T_, except for 7.95. The mean length of early veliger larvae was 504.33 ± 92.64 μm, and pH had no significant effect on the time taken for embryos to reach the early veliger stage (SLR, *R* ^2^ = 0.099, *F*(1, 17) = 1.764, *p* = 0.203). There was no difference in the average early veliger length among pH treatments (one‐way ANOVA, *F*(7, 17) = 0.625, *p* = 0.726; Figure 4C). Veliger larvae were first recorded in the samples between the ninth‐ and twentieth DPS (14.06 ± 2.61 DPS) with a mean length of 825.88 ± 106.92 μm. pH had no significant effect on the time taken for embryos to reach the veliger stage (SLR, *R* ^2^ = 0.0008, *F*(1, 37) = 0.028, *p* = 0.867), nor on the average veliger length among pH treatments (one‐way ANOVA, *F*(8, 37) = 1.330, *p* = 0.268; Figure 4D). After reaching the veliger stage, a notable difference in development was observed in pH_T_ 7.51–7.22. The veliger stage lasted until the 22nd DPS in pH_T_ 7.95–7.67, while in the lower pH_T_, viable veliger larvae were sampled until the 32nd DPS with no further change in size (Figure 5).

Pediveliger was not observed in pH_T_ 7.42–7.22, although there was one spawn per pH_T_ that reached hatching. Presented data for pediveliger larvae range from pH_T_ 7.95–7.51. The pediveliger stage was reached on average 27.23 ± 4.12 DPS. The mean pediveliger length was 1133.05 ± 83.27 μm. There was no significant effect of pH on the average pediveliger length (one‐way ANOVA, *F*(5, 24) = 0.895, *p* = 0.504; Figure 4E). pH had no significant effect on the time taken for embryos to reach the pediveliger stage (SLR, *R* ^2^ = 0.029, *F*(1, 24) = 0.682, *p* = 0.417). Hatching started on average on 31.46 ± 2.66 DPS with an average hatchling length of 1412.08 ± 112.85 μm. pH had no significant effect on the time when the hatching started (SLR, *R* ^2^ = 0.098, *F*(1, 28) = 2.955, *p* = 0.097) and there was no significant difference in the hatchling length among pH treatments (one‐way ANOVA, *F*(8, 27) = 0.482, *p* = 0.854; Figure 4F).

The spawns of banded‐dye murex in all pH treatments have reached trochophore, early veliger and veliger stage, except for one spawn from the control treatment (pH_T_ 7.95) that arrested at the beginning of embryonic development and was therefore excluded from further analysis. The proportion of spawns that have reached a certain developmental stage was calculated out of the initial number of spawns in each pH_T_. There was a decline in the number of spawns that reached the pediveliger and hatchling stage from pH_T_ 7.51–7.22. The binary logistic regression model applied to determine if pH had a significant effect on the likelihood of reaching the pediveliger and hatchling stage of development was statistically significant (*χ* ^2^(1) = 11.852. *p* < 0.001; *χ* ^2^(1) = 10.637, *p* = 0.001, respectively). For every 0.1 decrease in pH, a 0.001 decrease in the log‐odds of reaching the pediveliger stage is expected (*p* = 0.001, 95% CI [3.260E‐5, 0.059]), whereas log‐odds for reaching the hatchling stage decrease by 0.01 (*p* = 0.001, 95% CI [0.001, 0.160]).

### 3.3 Carryover Effect

**FIGURE 6:**
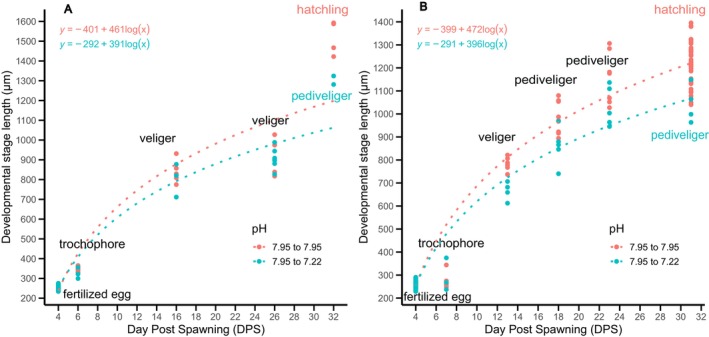
Relationship between the *Hexaplex trunculus* developmental stage length (μm), the developmental stage (trochophore, veliger, pediveliger, hatchling) and the day post‐spawning (DPS) for half of the spawn from pHT 7.95 (blue) and transplanted in pHT 7.22 (pink) from (A) female 1 & (B) female 3.

Spawns of banded‐dye murex from pH_T_ 7.95 (females F1, F3, & F6) were cut in half and maintained at their original pH or cross‐transplanted to pH_T_ 7.22. Intracapsular development was arrested at the trochophore stage in spawn from female F6, therefore it was excluded from further analysis. Overall, the spawns transplanted from pH_T_ 7.95 to 7.22 had a lower growth rate than spawns that remained in the pH_T_ 7.95 (LMM, *F*(1, 462) = 44.037, *p* = 0.001; MD = 25.90, SE = 6.385, *p* = 0.001). Both spawns in pH_T_ 7.95 have reached the hatchling stage, while the transplants in pH_T_ 7.22 only reached pediveliger (Figure 6). A negative effect of a low pH on the growth rate (in length, μm ln DPS) was observed in the spawn from female 3 (ANCOVA, *F*(1, 291) = 9.101, *p* = 0.003), with larvae transplanted in pH_T_ 7.22 having a lower mean length (MD = 23.83, SE = 7.90, *p* = 0.003). No significant difference in the growth rate between the treatments was observed for the spawn from female 1 (ANCOVA, *F*(1,169) = 0.634, *p = 0*.427).

**FIGURE 7:**
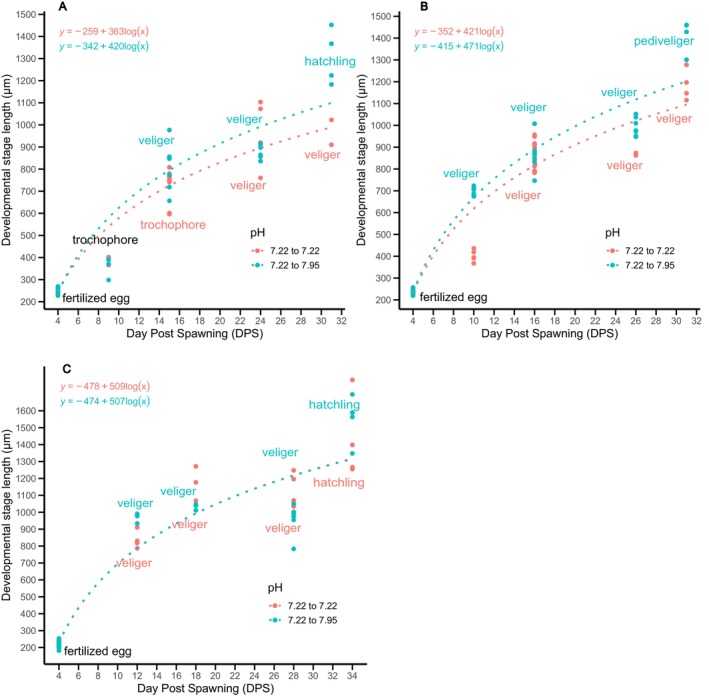
Relationship between the *Hexaplex trunculus* developmental stage length (μm), the developmental stage (trochophore, veliger, pediveliger, hatchling) and the day post‐spawning (DPS) for half of the spawn from pHT 7.22 (pink) and transplanted in pHT 7.95 (blue) from (A) female 4, (B) female 7 & (C) female 9.

Spawns from pH_T_ 7.22 (females F4, F7 and F9) were cut in half and maintained at their original pH or cross‐transplanted to pH_T_ 7.95. No significant difference was observed in the growth rate between the spawns transplanted to pH_T_ 7.95 and spawns that remained in 7.22 (LMM, *F*(1, 426) = 2.639, *p* = 0.105). Despite no observed difference in larval length, the spawns from females F4 and F7 in pH_T_ 7.22 reached the veliger stage and did not develop further, while their transplants in pH_T_ 7.95 reached the hatchling and pediveliger stage, respectively (Figure 7A,B). Only the spawn from female F9 reached the hatchling stage in both pH_T_ 7.22 and 7.95 (Figure 7C). An analysis of individual spawns and their respective pH treatments revealed a significant difference in growth rate between treatments only for F7 spawn (ANCOVA, *F*(2, 169) = 6.534, *p* = 0.001), with larvae transplanted in pH_T_ 7.95 having a higher growth rate than in pH_T_ 7.22 (MD = 28.186, SE = 11.027, *p* = 0.011).

**FIGURE 8:**
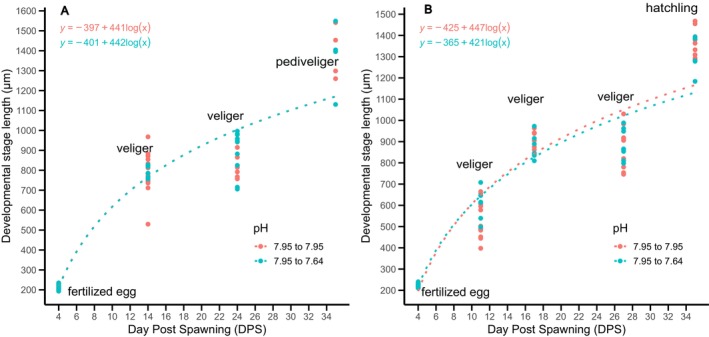
Relationship between the *Hexaplex trunculus* developmental stage length (μm), the developmental stage (trochophore, veliger, pediveliger, hatchling) and the day post‐spawning (DPS) for half of the spawn from pHT 7.95 (blue) and transplanted in pHT 7.64 (pink) from (A) female F6 & (B) female F8.

Spawns from females F8 and F10 in pH_T_ 7.95 were cut in half and maintained at their original pH or cross‐transplanted to pH_T_ 7.64. Intracapsular development from female 10 arrested at the veliger stage, and therefore was excluded from further analysis. Overall, no significant difference in growth rate was observed between the treatments (LMM, *F*(1, 252) = 0.033, *p* = 0.857). Spawn from female F6 reached the pediveliger stage in both pH_T_ 7.95 and 7.64 (Figure 8A), while the spawn from female 8 reached the hatchling stage in both pH_T_ (Figure 8B) with no observable difference between the growth rate (ANCOVA, *F*(1, 102) = 0.003, *p* = 0.959; *F*(1, 141) = 0.24, *p* = 0.877; respectively).

The spawns from females F4, F8, and F10 in pH_T_ 7.64 were cut in half and maintained at their original pH or cross‐transplanted to pH_T_ 7.95. Larvae from female F8 had arrested development at the veliger stage and were excluded from the further analysis. There was a significant difference in the growth rate between treatments (LMM, *F*(1,289) = 9.381, *p* = 0.002).

**FIGURE 9:**
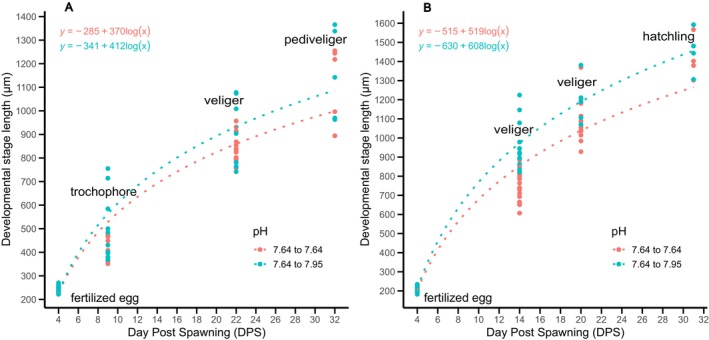
Relationship between the *Hexaplex trunculus* developmental stage length (μm), the developmental stage (trochophore, veliger, pediveliger, hatchling) and the day post‐spawning (DPS) for half of the spawn from pHT 7.64 (blue) and transplanted in pHT 7.95 (pink) from (A) female F4 & (B) female F10.

Transplants in both pH_T_ 7.64 and 7.95 from females F4 and F10 reached the pediveliger and hatchling stage, respectively (Figure 9A,B). A significant difference between treatments for the growth rate was observed only for the spawn from female 10, with larvae at pH_T_ 7.95 having a higher growth rate than at 7.64 (ANCOVA, *F* (1, 167) = 18.251, *p* = 0.001; MD = 54.196, SE = 12.686, *p* = 0.001).

Due to the observed variability in the larval growth rate between spawn at the same pH treatment, an effect size relative to the control pH_T_ was calculated to further assess the magnitude of the difference in intracapsular growth between treatments:

$$\text{Relative effect size}=-\left(\text{growth}\;{\text{rate}}_{\left(\mathrm{pHT}\;\text{parent acclimation}\right)}-{\text{growth rate}}_{\left(\mathrm{pHT}\;\text{transplanted}\right)}\right)/\text{growth}\;{\text{rate}}_{\left(\mathrm{pHT}\;\text{parent acclimation}\right)}$$

The pH_T_ parent acclimation were the treatments where the spawning occurred, while the pH_T_ transplanted were considered to be where the other half of the spawn was transferred. The effect size relative to the control was calculated for each replicate and plotted against the control growth rate. A positive effect size indicates that the embryo growth rate was higher in the transferred pH_T_ than the control, and a negative effect size indicates the opposite—the growth rate was lower in the transferred pH_T_ than the control.

Larvae with a higher growth rate in pH_T_ 7.95 were more affected when transferred to low pH, and larvae with a lower growth rate in pH_T_ 7.22 had higher growth rates when transferred to 7.95. For transplants between pH_T_ 7.95 and 7.64, the effect size was very small (between −0.05 and 0.09), and there was no observable relationship between the initial growth rate and the growth rate of the transplanted spawns.

## 4 Discussion

The reproductive cycle of *H. trunculus* is strongly dependent on temperature, as well documented by the start of the spawning periods of populations in different locations such as in Tunisia (early April, 22°C), Portugal (late April, 20°C), or Turkey (May, 19°C) (Vasconcelos et al. 2004; Lahbib et al. 2009; Güler and Lȍk 2014). In this study, banded‐dye murex began spawning 10 days after the temperatures reached 20°C, coinciding with the observations of spawning in the natural population in Bistrina Bay (personal observation). The spawning lasted about 3 days, a duration reported in previous studies (Vasconcelos et al. 2004; Lahbib et al. 2010). The spawned capsules were slightly smaller than in other studied populations. However, the size of the capsules has previously been reported to be highly variable, presumably due to different environmental factors (Lahbib et al. 2010). *H. trunculus* population from northern Tunisia had a smaller average capsule size (Lahbib et al. 2009, 2010) than populations from the Aegean Sea (Güler and Lȍk 2014) and Portugal (Vasconcelos et al. 2004). The average number and size of eggs in this study is consistent with previous findings (Lahbib et al. 2009, 2010), and so are the observed sizes of each developmental stage from fertilized egg to hatchling (Lahbib et al. 2010; Güler and Lȍk 2014).

pH had no significant effect on these reproductive traits in our experiment. Statistical analyses confirmed no significant differences among treatments, and no subtle non‐significant trends (e.g., reduced fecundity or capsule number) were observed beyond the variation already reported. There are a variety of coping mechanisms that a species may employ to deal with low pH. There are examples of successful gametogenesis, but insufficient energy reserves for successful fertilization or hatching (Kimura et al. 2011), which may also be the case in this study where pH had no effect on the fertilization, spawning of *H. trunculus*, and the larval growth rates but showed a delay in development for the late stages. In pH_T_ 7.95 to 7.51, veliger larvae continued to develop into pediveliger larvae. However, at lower pH (pH_T_ 7.42–7.22), the number of spawns that reached pediveliger decreased significantly. Delay in development was observed with viable veligers being sampled until the 32nd day post‐spawning with no further change in size, and fewer than 25% of spawns managed to develop to the hatchling stage. The magnitude of developmental delays and reduced viability observed in *H. trunculus* is comparable to effects reported in other gastropods and marine invertebrates (Ellis et al. 2009; Montroy et al. 2016; Hu et al. 2018), although less severe than total reproductive failure seen in some barnacles (Pansch et al. 2018).

Early life stages are generally considered more sensitive to ocean acidification than later stages, likely due to higher energy requirements to maintain homeostasis (Stumpp et al. 2011; Bergman et al. 2018; Kriefall et al. 2018; Lee et al. 2020). However, parental exposure to stressful conditions may influence offspring resilience or sensitivity (Chirgwin et al. 2018; Lee et al. 2020). For example, while elevated *p*CO_2_ had a negative effect on the oyster larvae *S. glomerata* , preconditioning the parent generation to elevated *p*CO_2_ resulted in a positive carryover effect on larvae in the form of larger and faster‐developing larvae, even though the larvae of both parent generations had similar survival rates (Parker et al. 2010). The phenotypic characteristics of another oyster larva, *C. hongkongensis*, also improved following parental exposure to low pH (Lim et al. 2021). In contrast, parental exposure to low pH had no effect on the sea anemone *N. vectensis* offspring's performance, but it did affect the parental gamete production and physiology (Glass et al. 2023).

In this study, the intracapsular development of *H. trunculus* larvae preconditioned to ambient pH_T_ was negatively affected by low pH_T_ 7.22, as evidenced by a reduced ability to reach the hatching stage, unlike half of the spawn that remained in the ambient. Exposure of the parents to pH_T_ 7.22 had even more pronounced negative effects on the larvae, which mostly developed to the veliger stage. These results align with reports of negative carryover effects in previous studies, such as reduced larval survival in the sea urchin *P. lividus* (Marčeta et al. 2022), reduced larval size and impaired development in the sea star *Asterias rubens* Linnaeus, 1758 (Hu et al. 2018), and negative effects on overall fitness in the North Atlantic bivalves *Mercenaria mercenaria* (Linnaeus, 1758) and *A. irradians* (Griffith and Gobler 2017).

These differences in carryover effects depend primarily on the mechanisms employed by the parent generation to cope with ocean acidification. Negative carryover effects observed in this study could be due to a reduced energy transfer between the parent generation and their offspring, as observed in common sea star *A. rubens* where parental pre‐acclimation to low pH negatively affected the larval size and development (Hu et al. 2018). When organisms are exposed to low pH, their energy requirements to maintain homeostasis increase, which could result in less energy being provided for gametogenesis (Pörtner and Farrell 2008). While this did not affect the reproductive performance or egg size of *H. trunculus* in this study, it could have affected the egg's nutritional quality (Allen et al. 2008). For example, in two abalone populations, a relationship between larval performance and maternal provisioning was demonstrated, with the maternal provisioning of lipids to offspring varying across populations and a positive correlation between lipid concentrations and survival at low pH (Swezey et al. 2020). *H. trunculus* larvae from only one spawn exposed to parental pH_T_ 7.22 managed to hatch in the same pH_T_ and did not show significant differences in length compared to the larvae hatched in the control group, indicating variability in response within the population. In addition, the larvae from the spawn preconditioned to pH_T_ 7.22 and transferred to ambient pH demonstrated that a negative effect could be reversed by reaching either the pediveliger or the hatching stage. This suggests that removal from stressful conditions leaves larvae with more available energy for the development of larval structures, likely because less energy is expended on maintaining the acid–base balance (Byrne 2011). In contrast to the ability to reach a particular developmental stage, the effect on average length between the spawns was much more variable, showing either positive or negative effects or no effect at all for each pH transplant. The differential responses of spawns indicate a possible interplay between parental exposure and offspring sensitivity, as well as a stronger influence of parental exposure on certain individuals. Intraspecific variation in response to ocean acidification has already been reported for several species (Kurman et al. 2017; Sekizawa et al. 2017; Kurihara et al. 2018), highlighting the importance of considering variability when assessing a species' ability to persist. For example, both intra‐ and interspecific variation in calcification and photosynthetic efficiency was observed in two branching corals, *Montipora digitata* (Dana, 1846) and *Porites cylindrica* Dana, 1846 (Sekizawa et al. 2017). Within‐population variability in this study was supported by the relative effect size, which showed that the better the larval performance was in pH_T_ 7.95, the more it worsened when transferred to pH_T_ 7.22, while larvae with poorer performance in pH_T_ 7.22 thrived better when transplanted to pH_T_ 7.95. Smith et al. (2019) observed similar changes in the fertilization success rate of two sea urchins, *Lytechinus pictus* (Verrill, 1867) and *Heliocidaris erythrogramma* (Valenciennes, 1846), under ambient and future pH conditions. In their study, the individuals with high fertilization success under the current pH conditions had a significantly lower success at low pH, while the individuals with low fertilization success actually improved their performance. If there is sufficient genetic variability within the population of a given species, adaptive responses to ocean acidification could be aided by profiling genotypes that are more resilient than others (Kurman et al. 2017).

The effect of parental exposure may depend on the specific pH conditions. In this study, the larvae from parents preconditioned to ambient pH_T_ 7.95 showed no difference in their growth rate and ability to reach the developmental stage (pediveligers and hatchlings) when transferred to 7.64. The lowest reported pH_T_ in Bistrina Bay, the natural habitat of the studied population, was pH_T_ 7.73, so the banded‐dye murex is likely able to cope with 0.1 units lower pH in the short term. The larvae of the parents preconditioned to pH_T_ 7.64 showed different responses, with the larvae of one spawn again demonstrating no difference whether they developed in 7.64 or were transferred to the ambient. In contrast, the larvae of the other spawn grew significantly faster at pH_T_ 7.95 (even faster than the larvae of the parents that were conditioned to pH 7.95), although the transplanted spawns hatched at both pHs. Small effect sizes suggest that the effect of the parental exposure on offspring sensitivity is subtle in the pH_T_ 7.64 transplants. The subtlety of the effect could be due to the moderate pH change, and larger effect sizes in certain cases (such as the female 10 spawn) highlight the importance of individual variation. This suggests that a pH of 7.64 is not detrimental to the banded‐dye murex, and that it already has coping mechanisms to deal with it, although variability within populations in terms of growth is present as it might already be close to its tipping point. *H. trunculus* populations from the Adriatic Sea have already been shown to be significantly genetically and epigenetically differentiated (Šrut et al. 2023), suggesting that the observed differences could appear due to different genotypes or—more likely—explained by variation in phenotypic plasticity traits expressed through epigenetic mechanisms. However, the possible presence of tolerant genotypes suggests that *H. trunculus* has a potential for adaptation (Foo et al. 2012). Notably, our experiment did not include juvenile or post‐hatch stages, but the marked variability among spawns suggests that different life stages may respond differently. Stage‐specific assessments will be important to fully understand resilience across the life cycle. Given the ecological role of *H. trunculus* as a key predator of bivalves, reduced larval success under acidified conditions could alter benthic community dynamics and indirectly affect shellfish aquaculture in the Mediterranean. These findings underscore the importance of considering OA impacts on predator–prey interactions when developing management strategies for coastal ecosystems.

Limitations of this study include the relatively small number of spawns analyzed, the focus on a single population, and the absence of post‐hatch juvenile monitoring. Future studies should incorporate multi‐population comparisons, larger sample sizes, and long‐term tracking of juvenile performance to better assess the generality of these results and the adaptive potential of *H. trunculus*.

Overall, these results highlight how the inclusion of natural pH variability, a broader range of pH conditions, and the measurement of different endpoints improve our ability to assess the potential resilience of a species in a given habitat. This study also contributes to the existing knowledge gap of intraspecific variation in response and emphasizes the importance of considering individual variability and parental influence on specific individuals when assessing species' sensitivity.

## Author Contributions

**Sanja Grđan:** conceptualization (lead), formal analysis (lead), investigation (lead), writing – original draft (lead), writing – review and editing (equal). **Sam Dupont:** conceptualization (supporting), supervision (equal), validation (lead), writing – review and editing (equal). **Luka Glamuzina:** data curation (lead), investigation (supporting), writing – review and editing (equal). **Ana Bratoš Cetinić:** conceptualization (supporting), resources (lead), supervision (equal), writing – review and editing (equal).

## Conflicts of Interest

The authors declare no conflicts of interest.

## Data Availability

The data that support the findings of this study are openly available in PANAGEA Data Publisher for Earth and Environmental Science at https://doi.org/10.1594/PANGAEA.983084.
